# Candidemia among Iranian Patients with Severe COVID-19 Admitted to ICUs

**DOI:** 10.3390/jof7040280

**Published:** 2021-04-08

**Authors:** Amir Arastehfar, Tahmineh Shaban, Hossein Zarrinfar, Maryam Roudbary, Mona Ghazanfari, Mohammad-Taghi Hedayati, Alireza Sedaghat, Macit Ilkit, Mohammad Javad Najafzadeh, David S. Perlin

**Affiliations:** 1 Center for Discovery and Innovation, Hackensack Meridian Health, Nutley, NJ 07110, USA; a.arastehfar.nl@gmail.com; 2 Department of Parasitology and Mycology, Faculty of Medicine, Mashhad University of Medical Sciences, Mashhad 9199-91766, Iran; shabanT981@mums.ac.ir; 3 Allergy Research Center, Mashhad University of Medical Sciences, Mashhad 91766-99199, Iran; h.zarrin@gmail.com; 4 Department of Parasitology and Mycology, School of Medicine, Iran University of Medical Sciences, Tehran 1449614535, Iran; m_roudbary@yahoo.com; 5 Invasive Fungi Research Center, Communicable Diseases Research Institute, Mazandaran University of Medical Sciences, Sari 48157-33971, Iran; ghazanfarimona71@gmail.com (M.G.); hedayatimt@gmail.com (M.-T.H.); 6 Department of Medical Mycology, Faculty of Medicine, Mazandaran University of Medical Sciences, Sari 48157-33971, Iran; 7 Lung Diseases Research Center, Faculty of Medicine, Mashhad University of Medical Sciences, Mashhad 9137913316, Iran; sedaghatar@mums.ac.ir; 8 Division of Mycology, University of Çukurova, Adana 01330, Turkey; macitilkit@gmail.com

**Keywords:** COVID-19, COVID-19-associated candidemia, multidrug resistance, *Candida albicans*, *Candida glabrata*, *Rhodotorula mucilaginosa*

## Abstract

As a novel risk factor, COVID-19 has led to an increase in the incidence of candidemia and an elevated mortality rate. Despite being of clinical importance, there is a lack of data regarding COVID-19-associated candidemia (CAC) among Iranian patients. Therefore, in this retrospective study, we assessed CAC epidemiology in the intensive care units (ICUs) of two COVID-19 centers in Mashhad, Iran, from early November 2020 to late January 2021. Yeast isolates from patients’ blood were identified by 21-plex polymerase chain reaction (PCR) and sequencing, then subjected to antifungal susceptibility testing according to the CLSI M27-A3 protocol. Among 1988 patients with COVID-19 admitted to ICUs, seven had fungemia (7/1988; 0.03%), among whom six had CAC. The mortality of the limited CAC cases was high and greatly exceeded that of patients with COVID-19 but without candidemia (100% (6/6) vs. 22.7% (452/1988)). In total, nine yeast isolates were collected from patients with fungemia: five *Candida albicans*, three *C. glabrata*, and one *Rhodotorula mucilaginosa*. Half of the patients infected with *C. albicans* (2/4) were refractory to both azoles and echinocandins. The high mortality of patients with CAC, despite antifungal therapy, reflects the severity of the disease in these patients and underscores the importance of rapid diagnosis and timely initiation of antifungal treatment.

## 1. Introduction

Critically ill patients with COVID-19 admitted to intensive care units (ICUs) are at an increased risk of developing secondary infections caused by bacterial and fungal pathogens [1,2,3]. Indeed, several studies have reported a high mortality rate for patients with COVID-19 co-infected with pathogenic fungi, both filamentous and yeast species [2,3]. An increasing body of evidence points to a higher incidence of candidemia in the post-COVID-19 than in the pre-COVID-19 era [4,5,6]. The mortality rate among patients with COVID-19-associated candidemia (CAC) is high [5,6,7,8,9], reaching up to 83% despite antifungal treatment [8]. Further complicating matters is the emergence of antifungal drug-resistant *Candida* isolates [7,8,10], especially pan-echinocandin-resistant *C. glabrata* [10] and multidrug-resistant *C. auris* [7,8], which may potentially cause therapeutic failure, unfavorable clinical outcomes, and outbreaks. Therefore, a clear understanding of the pathology, epidemiology, and antifungal treatment of CAC is of paramount importance for clinical success and the reduction of mortality rates.

Although Iran was among the first countries to be severely hit by the COVID-19 pandemic, the data regarding the occurrence of CAC in Iran are lacking. Here, we report the results of a retrospective study in which the epidemiology of CAC was analyzed among patients with severe COVID-19 admitted to two hospitals in Mashhad, south-eastern Iran. Our study revealed a different epidemiological picture and a higher mortality in patients with COVID-19 and candidemia compared to those with COVID-19 only, which underscores the need for prompt diagnosis and treatment of CAC.

## 2. Materials and Methods

### 2.1. Patient Population, Diagnosis, and Treatment

All critically ill patients with COVID-19 admitted to COVID-19 ICUs of Ghaem and Imam Reza hospitals in Mashhad, from early November 2020 to late January 2021, were retrospectively evaluated for candidemia. COVID-19 was diagnosed based on positive real-time polymerase chain reaction (PCR) tests for SARS-CoV-2 and candidemia was identified by *Candida*-positive blood cultures. This was a non-interventional study, and the authors did not have any influence on the prescription of antifungal treatment. Patients treated with fluconazole or caspofungin received initial (loading) doses of 800 or 70 mg on the first day, which were de-escalated to 400 or 50 mg/day from the next day onwards, respectively. Colonies obtained from positive blood cultures were grown on Sabouraud dextrose agar and chromogenic agar to reveal phenotypic characteristics and ensure the purity of the obtained colonies. This study was approved by the ethical committee of the Mashhad University of Medical Sciences (ethical approval number IR.NIMAD.REC.1398.103, 16 June 2019).

### 2.2. DNA Extraction and Species Identification

DNA was extracted using the cetyl trimethylammonium bromide-based method [11]. Because there was no immediate access to Sanger sequencing, all isolates were primarily identified by using a multiplex 21-plex PCR assay [12,13], which is used routinely in our research setting, and the classification was confirmed by internal transcribed spacer (ITS) sequencing with ITS1 and ITS4 primers [14].

### 2.3. Antifungal Susceptibility Testing (AFST)

AFST was performed following the Clinical Laboratory Standards Institute (CLSI) M27-A3 protocol [15]. Fluconazole, voriconazole, itraconazole, amphotericin B (AMB) (all from Sigma-Aldrich, St. Louis, MO, USA), caspofungin (bioMérieux SA, Marcy-l’Étoile, France), and anidulafungin (Pfizer, New York, NY, USA) were included in AFST. *C. parapsilosis* (ATCC 22019) and *C. krusei* (ATCC 6258) type strains were used for quality control. Isolates were seeded on the plates containing antifungal drugs and incubated at 35 °C for 24 h. Minimum inhibitory concentrations (MICs) were determined visually and interpreted based on the available clinical break points and epidemiological cut-off values according to the CLSI-M60 document [16] and previously established definitions [17]. *C. albicans* and *C. glabrata* isolates responding to fluconazole MICs ≥8 and ≥64 µg/mL or to echinocandin MICs ≥1 and ≥0.5 µg/mL, respectively, were considered resistant to these drugs [17]. MICs of AMB, voriconazole, and itraconazole were interpreted based on epidemiological cut-off values; *C. albicans* and *C. glabrata* isolates showing AMB MICs >2 µg/mL, itraconazole MICs >0.12 and >2 µg/mL, and voriconazole MICs >0.03 and >0.5 µg/mL, respectively, were considered non-wild-types [17]. Moreover, the clinical breakpoints of voriconazole and itraconazole were also available for *C. albicans*, and isolates with MICs ≥ 1 µg/mL were regarded as resistant to these antifungals [17]. AFST was performed in a retrospective manner after the study period was terminated since it is not performed routinely in clinical care settings.

### 2.4. FKS1 Sequencing

Hotspot 1 (HS1) and Hotspot 2 (HS2) of *FKS1* were sequenced for echinocandin-resistant *C. albicans* isolates using the primers, PCR, and sequencing conditions described previously [18]. The raw sequence data were edited using SeqMan Pro (DNASTAR, Madison, WI, USA), edited sequences were aligned using MEGA v7.0 (Temple University, Philadelphia, PA, USA) [19], and *C. albicans* ATCC 32354 served as the wildtype [18].

### 2.5. Statistical Analysis

SPSS software (v24 for Windows; SPSS Inc., Chicago, IL, USA) was used for statistical analysis. The mortality rates between patients with CAC and those with COVID-19 but without candidemia were compared by a *t*-test.

### 2.6. Availability of Sequence Data

Sequence data of HS1 and HS2 of *FKS1* were deposited in NCBI and GenBank accession numbers were assigned for respective isolates as follows: MW847604-MW847605 and MW847606-MW847607, respectively.

## 3. Results

### 3.1. Patients’ Characteristics

In total, 1988 critically ill patients with COVID-19 were treated in ICUs of Ghaem and Imam Reza hospitals during the three-month study period; among them, seven patients had fungemia (0.03%), among whom six had candidemia (6/7; 85.7%). Overall, nine isolates were obtained from the seven patients with fungemia, as two patients (P110 and P121) carried two isolates each. The clinical profiles of patients with CAC are summarized in Table 1. The median age of the patients was 68 years (range 27–75 years); almost 72% of them (5/7) were older than 40 years and the rest were younger than 30 years. All patients had central venous catheters (CVCs) and received broad-spectrum antibiotics and total parenteral nutrition; approximately 72% of them (5/7) were mechanically ventilated. Almost 43% of the patients with fungemia (3/7) were suffering from cancer and previously received chemotherapy (one patient was also diabetic); the remaining 57% (4/7) did not have any underlying diseases. All patients with CAC were treated with antifungal drugs. Persistent candidemia was noted in two patients (P110 and P121). One patient (P112), who had fungemia due to a non-*Candida* species, was treated with fluconazole and his CVC was removed (Table 1). The median hospitalization time was 33.5 days (range 7–83 days). The mortality of patients with fungemia was 85.7% (6/7), which was significantly higher than that of critically ill patients with COVID-19 but without candidemia (22.8%; 452/1981). It is noteworthy that the mortality rate of patients with CAC, only considering patients with candidemia due to *C. albicans* and *C. glabrata*, was 100% (6/6).

### 3.2. Yeast Species Prevalence

All the species were correctly identified by 21-plex PCR as confirmed by ITS sequencing. *C. albicans* was the leading cause of CAC (4/7; 57.2%), followed by *C. glabrata* (2/7; 28.4%), and *Rhodotorula mucilaginosa* (1/7; 14.2%) (Table 1). Two patients with persistent candidemia, P110 and P121, carried two *C. albicans* and two *C. glabrata* isolates, respectively (Table 1). Accordingly, *C. albicans* represented 55.5% of yeast isolates (5/9), followed by *C. glabrata* (3/9, 33.3%), and *R. mucilaginosa* (1/9; 11.2%).

### 3.3. AFST and HS1–HS2 of FKS1 Sequencing

Among the patients infected with *C. albicans*, two (2/4; 50%) harbored fluconazole-resistant isolates (MICs ≥8 µg/mL), which were also resistant to echinocandins (MICs ≥1 µg/mL) (Table 2). These two patients (P123 and P124) were treated with fluconazole and caspofungin and both showed therapeutic failure. None of the *C. glabrata* isolates were resistant to the tested antifungal drugs. The *R. mucilaginosa* isolate showed high MICs of all azoles and echinocandins tested but a low MIC of AMB (Table 2).

Since two of our *C. albicans* isolates were resistant to both caspofungin and anidulafungin (#123 and #124), we performed HS1- and HS2-*FKS1* sequencing. Isolate #123 harbored a mutation in HS1 (T1922C) corresponding to F641S, while isolate #124 did not harbor any mutations.

## 4. Discussion

An increasing number of studies have documented a higher incidence of candidemia in the post-COVID-19 era and its association with high mortality despite antifungal treatment [3,4,5,6,7,8,9,20]. In this retrospective, multicenter study performed in Iran, we confirmed a high mortality rate among patients with CAC, which further highlights the vital importance of timely diagnosis and administration of proper antifungal treatment.

The risk factors of CAC observed in the current study were similar to those previously reported for candidemia: the use of broad-spectrum antibiotics, CVC insertion, mechanical ventilation, and cancer. The administration of the IL-6 inhibitor, tocilizumab, to mitigate the cytokine storm has been reported to promote candidemia among severely ill COVID-19 patients [21]; however, none of our patients received this medication.

Analysis of the epidemiological spectrum in our study population showed that *C. albicans* and *C. glabrata* were the leading causative agents of CAC. In contrast, our previous multicenter studies indicated that prior to 2019, *C. parapsilosis* was the main cause of candidemia in Mashhad, resulting in persistent outbreaks from 2015–2019 [22]; because, unlike *C. albicans* and *C. glabrata*, *C. parapsilosis* is usually thought to be acquired from external sources, this result prompted us to speculate about the environmental origin of infection and horizontal transfer. However, the present finding on the predominance of *C. albicans* and *C. glabrata* in patients with CAC supports the hypothesis that the disturbance of the host defense mechanisms caused by SARS-CoV-2, such as the breakdown of the epithelial barrier, along with other risk factors, promotes colonization and opportunistic infection of *Candida* spp. existing in the commensal state of the human microbiome [3]. Our previous studies focusing on the epidemiology of superficial candidiasis have also identified *C. albicans* and *C. glabrata* as the leading agents [23,24], which may reflect the predominance of these two species in the mycobiome of the Iranian population. In keeping with these observations and the hypothesis that the disruption of the gut epithelium due to COVID-19 facilitates the translocation of yeast to deep organs [3], a study in Greece found that critically ill patients with COVID-19 receiving *Saccharomyces cerevisiae*-containing probiotics developed fungemia caused by that yeast species [25]. The absence of *C. parapsilosis* among CAC-causative *Candida* may be attributed to a stricter infection control and environmental decontamination in the post-COVID-19 era; however, in the centers included in our study, the sanitation measures were less rigid and there was a surge of severe COVID-19 cases. The identification of *C. albicans* and *C. glabrata* as the leading agents of CAC is in line with the reports from Oman [9], Italy [21], and the United Kingdom [6,20]; still, this result does not rule out the possibility that other *Candida* spp. may cause outbreaks among critically ill patients with COVID-19, as evidenced by the finding that *C. auris* was the main causative species of CAC in India [7], Mexico [8], and Brazil [26]. Altogether, the epidemiological profile of patients with CAC observed in this study may reflect the endogenous rather than nosocomial route of *Candida* infection, whereas the single case of *R. mucilaginosa* infection could have an environmental origin.

Drug-resistant isolates responsible for CAC have been reported to belong to *C. glabrata* and *C. auris* [7,8,10]. Moreover, analysis of the worldwide collection of *Candida* blood isolates recovered over the course of 20 years indicates that *C. albicans* is the species with the lowest rate of drug resistance [27]. Yet, in this study none of the *C. glabrata* isolates were drug-resistant, whereas 50% of the patients infected with *C. albicans* harbored isolates resistant to both azoles and echinocandins and were treated with fluconazole followed by caspofungin, which ultimately led to therapeutic failure. Thus, our study is the first to identify multidrug-resistant *C. albicans* among patients with CAC. When sequencing FKS1, we found that one of the *C. albicans* isolates carried F641S, which has been identified previously [18], while the other isolate did not harbor any mutations in neither HS1 nor HS2. Consistent with other studies, the only *R. mucilaginosa* isolate detected here showed high MICs of azoles and echinocandins [28,29], but the infected patient survived after CVC removal and fluconazole treatment.

With the exception of the one *R. mucilaginosa-* infected patient, all patients with CAC died despite treatment with antifungal drugs, indicating an extremely high mortality rate, which significantly exceeded that of patients with COVID-19 but without candidemia (100% (6/6) vs. 22.7% (452/1988)). We should note that the high mortality reported here cannot be solely attributed to candidemia but is more likely to be a factor further complicating the severity of the disease of patients who were severely ill and already admitted for intensive care (it may well be a marker for terminal stage disease). Nonetheless, it was still much higher than the mortality rates reported from other multicenter candidemia studies conducted in Iran [22,30,31]. Although the mortality observed in this study is similar to that reported in Mexico (83%) [8], it is much higher than those documented in many other studies [3,5,6,7,8,9,10,25]. It is noteworthy that rapid diagnostic serology tools are not widely used in Mashhad because of their high cost [32], and physicians start antifungal treatment only after positive blood culture tests. Therefore, the exceptionally high mortality rate revealed here could be attributed to late diagnosis and delayed treatment of patients with fluconazole rather than echinocandins as the first-line antifungal drugs. The preferred use of fluconazole as the first-line antifungal medication is driven by the higher cost of echinocandins, which seems to be a common issue in developing countries [22,30,31,33,34]. Therefore, our results emphasize the importance of prompt diagnosis and timely initiation of proper antifungal treatment to achieve clinical success and decrease mortality.

The limited number of CAC cases and the lack of detailed clinical data due to the retrospective nature of the current study were the main limitations of this study. Furthermore, because of the small number of available CAC cases, we could not perform appropriate mathematical and statistical assessment of risk factors that may have facilitated the development of candidemia. Therefore, detailed prospective large-scale studies on CAC need to be conducted in Mashhad in the future.

In conclusion, our study revealed a high mortality rate among critically ill patients with COVID-19 and candidemia in Iran, thus underscoring the importance of rapid diagnosis followed by the timely initiation of appropriate antifungal therapy.

## Figures and Tables

**Table 1:** Clinical profiles of patients with COVID-19-associated candidemia admitted to COVID-19 intensive care units of two hospitals in Mashhad, Iran, from November 2020 to late January 2021.

Patient #	Age/Sex	CVC	BA	PTN	MV	ID	Surgery	UD	HD (Days)	DTCD	Yeast Species (Isolate #)	AFT *	Outcome
**P94**	70/F	Yes	Yes	Yes	Yes	No	Brain surgery	Hematological malignancy	56	51	*C. glabrata* (#94)	Fluconazole→Caspofungin	Died
**P98**	40/M	Yes	Yes	Yes	Yes	No	No	None	83	2 7	*C. albicans* (#98)	Fluconazole	Died
**P110**	28/F	Yes	Yes	Yes	Yes	No	No	None	45	6	*C. albicans* (#110)	Fluconazole→Caspofungin	Died
											*C. albicans* (#111)		
**P112**	68/F	Yes	Yes	Yes	Yes	No	No	None	6	31	*R. mucilaginosa* (#112)	CVC removal + Fluconazole	Survived
**P121**	69/F	Yes	Yes	Yes	Yes	No	No	Endocervical cancer, diabetes	22	4	*C. glabrata* (#121)	Fluconazole→Caspofungin	Died
											*C. glabrata* (#122)		
**P123**	27/M	Yes	Yes	Yes	No	No	No	None	17	5	*C. albicans* (#123)	Fluconazole→Caspofungin	Died
**P124**	75/M	Yes	Yes	Yes	Yes	No	No	Ovarian cancer	7	1	*C. albicans* (#124)	Fluconazole→Caspofungin	Died

* The loading doses for fluconazole and caspofungin, 800 and 70 mg/day, were de-escalated to 400 and 50 mg/day, respectively. The data on the duration of antifungal treatment were not available. CVC, central venous catheter; BA, broad-spectrum antibiotics; PTN, parenteral nutrition; MV, mechanical ventilation; ID, immunosuppressive drug; HD, hospitalization duration; UD, underlying diseases; DTCD, days to candidemia diagnosis; AFT, antifungal treatment.

**Table 2:** Antifungal susceptibility profiles of yeasts recovered from patients with COVID-19-associated fungemia.

Yeast Species (Isolate #)	Minimum Inhibitory Concentration (µg/mL)					
	Fluconazole	Voriconazole	Itraconazole	Anidulafungin	Caspofungin	Amphotericin B
*C. glabrata* (#94)	8	0.032	0.25	0.25	0.25	0.5
*C. albicans* (#98)	0.5	0.016	0.06	0.06	0.06	0.125
*C. albicans* (#110)	0.125	0.016	0.25	0.016	0.016	0.5
*C. albicans* (#111)	0.125	0.016	0.125	0.016	0.016	0.5
*R. mucilaginosa* (#112)	64	2	8	4	4	0.03
*C. glabrata* (#121)	2	0.03	0.25	0.125	0.125	0.5
*C. glabrata* (#122)	4	0.03	0.25	0.25	0.25	0.5
*C. albicans* (#123)	64	16	16	1	2	1
*C. albicans* (#124)	8	0.125	4	1	1	1

## Data Availability

Sequence data of HS1 and HS2 of *FKS1* were deposited in NCBI and GenBank accession numbers were assigned for respective isolates as follows: MW847604-MW847605 and MW847606-MW847607, respectively.
